# Perioperative anaphylaxis with no identifiable cause

**DOI:** 10.1186/s40981-024-00688-2

**Published:** 2024-01-23

**Authors:** Tatsuo Horiuchi, Tomonori Takazawa

**Affiliations:** 1 Department of Anesthesiology, Gunma University Graduate School of Medicine, 3-39-22 Showa-machi, Maebashi, 371-8511 Japan; 2 Department of Anesthesiology, Faculty of Medicine, University of Toyama, 2630 Sugitani, Toyama, 930-0194 Japan

We read with great interest the paper written by Ida and colleagues, in which anesthesia for coronary artery bypass grafting for triple-vessel coronary artery disease was complicated by severe anaphylaxis requiring cardiopulmonary resuscitation. However, the authors could not identify the cause of the anaphylaxis despite skin tests and basophil activation tests (BATs).

The diagnosis of anaphylaxis is based on a combination of clinical symptoms and in vitro data, including serum tryptase levels [1]. In recent years, an objective tool for assessing the likelihood of anaphylaxis occurrence, called the Hypersensitivity Clinical Scoring Scheme (HCSS), has been developed and has been recommended for diagnosis [2]. The score for the case reported by Ida et al. was 21, indicating a high probability of the occurrence of anaphylaxis. However, serum tryptase concentration was measured only once, although it ideally should have been measured at least twice, since some patients, such as those with mastocytosis, have high tryptase concentrations even at regular times [3].

Although there might indeed be cases of anaphylaxis with no known cause even after post-allergy workup, such cases are rare. The Japanese Epidemiological Study for Perioperative Anaphylaxis (JESPA), a recent large Japanese study, reported that of 43 cases diagnosed with anaphylaxis, the cause could not be identified in 11 cases. Among the 43 cases, while neither the skin test nor the basophil activation test could be performed in four of them, the cause could not be identified in seven of the remaining 39 patients (18%) despite performing at least one of the tests [4]. The reasons for not finding the cause of anaphylaxis could be that the patient was not anaphylactic or that the test was a false negative. The latter was more likely in Ida et al.’s case. If the prick test is negative for all suspect drugs, a more sensitive intradermal test should be performed. Even if the dermatologist refuses to perform the intradermal test, the anesthesiologist should consider performing the test instead, as recommended in the Practical Guide of the Japanese Society of Anesthesiologists [1], and the risk of anaphylaxis from intradermal testing should not be overestimated. Further, the results of BATs should only be trusted when they are performed at proven and trusted institutions. Confirming the elevation of basophil activation markers in positive controls is essential when performing BATs.

Failure to identify the cause of anaphylaxis is a disadvantage for both the patient and the anesthesiologist. For example, anesthesiologists may have fewer drug options if the patient needs to undergo surgery in future, and the forced change in the procedure might increase the surgical invasiveness for the patient. Especially when rocuronium is the suspected drug but cannot be ruled out as the cause, as in this case, this is a serious problem. The only muscle relaxants available in Japan are rocuronium and suxamethonium; antigen cross-reactivity has been reported between them [5]. Although the drug is not currently available in Japan, the incidence of anaphylaxis caused by cisatracurium is about 1/40th that of rocuronium [6], and there is almost no antigenic cross-reactivity between rocuronium and cisatracurium [5]. Additionally, cisatracurium can be used as an alternative if the supply of rocuronium becomes unstable. We, thus, hope that it will soon become available in Japan.

In conclusion, anesthesiologists should make every effort to identify the cause of perioperative anaphylaxis due to the potentially serious outcomes of not knowing the cause.

## Data Availability

Not applicable.
